# Multifunctional Eutectic Mixtures Enable Integrated Recovery of U, Pu, and Sr From Nuclear Waste

**DOI:** 10.1002/advs.75174

**Published:** 2026-04-03

**Authors:** Yifan Wang, Yanjun Lv, Qilong Tang, Huaixin Hao, Jianfeng Jia, Gang Ye, Jing Chen, Chao Xu, Zhipeng Wang

**Affiliations:** ^1^ Institute of Nuclear and New Energy Technology Tsinghua University Beijing China; ^2^ College of Sciences Northeastern University Shenyang China

**Keywords:** integrated recovery, multifunctional eutectic mixtures, nuclear waste treatment, reverse micellar supramolecular structures

## Abstract

Addressing the challenges of nuclear waste management and strategic resource recovery necessitates novel functional materials for the integrated and efficient capture of critical elements. This work reports a class of multifunctional eutectic mixtures (EMs) constructed from crown ether (hydrogen‐bond acceptor, HBA) and partially esterified organophosphorus acids (hydrogen‐bond donor, HBD). This design integrates the high selectivity of crown ether toward Sr(II) with the strong affinity of organophosphorus ligands for U(VI)/Pu(IV), achieving unprecedented single‐step extraction efficiencies exceeding 99.9% for U(VI), 99.8% for Pu(IV), and 99% for Sr(II) under extreme and nearly realistic conditions. This exceptional performance originates from the differentiated metal coordination of crown ether and phosphoric esters, coupled with a synergistic “coordination‐encapsulation” effect mediated by the formation of reverse micellar supramolecular structures. Our findings not only provide high‐performance materials for the simultaneous recovery of critical radionuclides but also demonstrate the great potential of EMs as a sustainable platform for addressing complex separation scenarios.

## Graphical abstract

A novel class of multifunctional eutectic mixtures, crafted from crown ether and organophosphorus molecules, achieves highly efficient, simultaneous recovery of uranium, plutonium, and strontium from nuclear waste. The material operates via a dual “coordination‐encapsulation” mechanism within self‐assembled reverse‐micelle structures, offering a sustainable, one‐step solution for nuclear resource recycling and waste minimization.

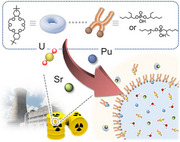

## 1 Introduction

Nuclear energy, characterized by its high energy density, stable power output, and clean low‐carbon footprint, has attracted widespread international attention in the context of global energy transition and environmental protection. However, the utilization of nuclear energy inevitably generates radioactive waste, whose safe treatment and disposal are crucial for the sustainable development of nuclear power [1, 2, 3, 4, 5]. Among the complex constituents of nuclear waste, uranium (U) and plutonium (Pu), which are both highly abundant and intensely radioactive, are critical nuclear materials with civilian and military dual‐use significance [2, 6, 7]. Meanwhile, the fission product strontium (Sr) poses a challenge to the safety of geological nuclear waste disposal due to its high heat generation [8, 9]. Its radioactive isotope, Sr‐90, also holds great application value in areas such as nuclear battery production and radiotherapy [10]. Therefore, the efficient recovery of U, Pu, and Sr from nuclear waste not only facilitates waste minimization and detoxification but also enables the recycling of strategically important resources, delivering dual environmental and resource benefits.

Currently, industrial‐scale separation and recovery of key elements from nuclear waste primarily rely on mature solvent extraction technologies, owing to their high throughput, continuous operation capability, and robust adaptability to extreme conditions (e.g., high acidity and radioactivity). Typically, ligands containing the P = O functional group (e.g., alkyl phosphoric acids/esters) are extensively employed for the extraction of U and Pu [11, 12, 13], while Sr capture is mainly achieved using crown ethers [9, 14]. However, this approach of applying different functional molecules and separation systems for multiple metals presents several practical challenges. First, the functional specificity of different extractants necessitates the design of multiple separation steps [15, 16, 17, 18, 19], increasing process complexity and operational costs. Second, the significant structural differences among functional molecules often require the use of different organic diluents [9, 20, 21], further amplifying system diversity and operational intricacy. Moreover, the integration of diverse separation units compromises process stability and controllability. Hence, optimization and improvement of existing processes are imperative.

The root cause of these issues lies in the relatively narrow functionality of traditional separation materials, which limits their ability to capture a broad spectrum of metal ions efficiently. Although designing integrated molecules could theoretically expand their functional scope, such strategies often encounter synthetic challenges and high preparation costs, hindering industrial adoption. In recent years, eutectic mixtures (EMs), a class of green solvent platforms formed through molecular self‐assembly between hydrogen bond acceptor (HBA) and hydrogen bond donor (HBD), have shown great promise in metal ion separation and resource recovery due to their tunability, low toxicity, biodegradability, and wide liquidus range [22, 23, 24, 25, 26]. More importantly, the composition and structure of EMs are highly designable. By rationally selecting and adjusting the types and ratios of HBA and HBD molecules, their physicochemical properties and coordination behaviors can be precisely tailored, enabling multifunctional integration within a single system. This allows for the one‐step, cooperative recovery of multiple target elements using a single solvent system. Furthermore, such functional materials eliminate the need for additional diluents, thereby simplifying the process flow and reducing material complexity, offering a promising solution to overcome the limitations of existing separation techniques.

Building on this rationale, we designed and constructed a series of novel EMs using a crown ether as the HBA and partially esterified organophosphorus acids (retaining P(= O)─OH groups) as the HBD. These EMs were designed to concurrently achieve efficient Sr capture and the deep recovery of U and Pu. The extraction behaviors toward U, Pu, and Sr were systematically evaluated, and the underlying mechanisms were elucidated through a combination of spectroscopic and interfacial characterization techniques. This work aims to provide a new material platform and theoretical foundation for advancing the functionalization and practical application of eutectic mixtures in nuclear waste treatment, thereby supporting the green upgrading of advanced nuclear fuel cycle systems.

## 2 Results and Discussion

### 2.1 Preparation and Characterization of EMs

**FIGURE 1:**
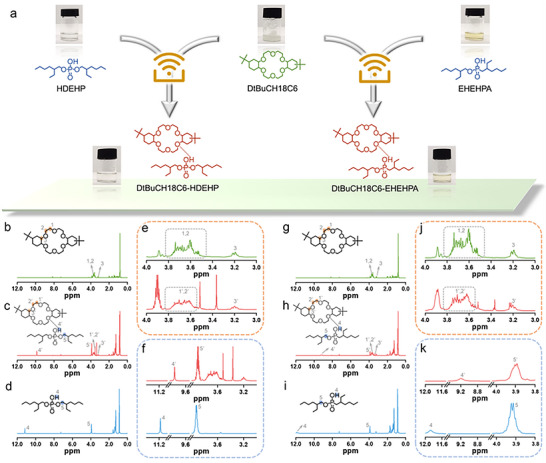
Preparation and characterization of EMs. (a) Structures and graphs of investigated HBA, HBD molecules, and the prepared EMs. ^1^H NMR spectra of (b) DtBuCH18C6, (c) DtBuCH18C6‐HDEHP EM (molar ratio 1/2), and (d) HDEHP. (e,f) Expanded spectral regions for comparative analysis. ^1^H NMR spectra of (g) DtBuCH18C6, (h) DtBuCH18C6‐EHEHPA EM (molar ratio 1/2), and (i) EHEHPA. (j,k) Expanded spectral regions for comparative analysis.

Recognizing that the types and molar ratios of HBA and HBD molecules can significantly influence the metal recovery behaviors of resulting EM materials, this study selected di‐*tert‐*butylcyclohexano‐18‐crown‐6 (DtBuCH18C6), a crown ether known for its exceptional selectivity toward Sr(II) ion, as the HBA [9, 14]. Two commercially available phosphoric esters, di‐(2‐ethylhexyl)phosphoric acid (HDEHP) and 2‐ethylhexylphosphoric acid mono‐2‐ethylhexyl ester (EHEHPA), both containing P(= O)‐OH functional groups, were chosen as HBDs [12, 27]. Through an ultrasound‐assisted method, we successfully constructed four distinct EMs with two different molar ratios (crown ether/phosphoric ester = 1/2 or 1/8). Notably, the originally solid paste‐state DtBuCH18C6 transformed into stable, homogeneous liquids with good fluidity upon mixing with the two phosphoric esters (Figure 1a), indicating the formation of eutectic mixtures with significantly depressed melting points via intermolecular hydrogen bonding between the selected HBA and HBDs [28, 29]. To elucidate the intermolecular interactions between the crown ether and phosphoric ester molecules, the synthesized EMs were systematically characterized using ^1^H NMR, ^13^C NMR, and FT‐IR spectroscopy. The results showed notable changes, including signal deformation or chemical shift variations, in the O‐C*H*_2_‐C*H*_2_‐O moiety of the crown ether, the characteristic P(= O)‐O*H* peak of the phosphoric esters, and the adjacent P‐O‐C*H*_2_‐ group upon EM formation (Figure 1b–k, Figures S1 and S2). These observations confirm the presence of significant hydrogen‐bonding interactions between DtBuCH18C6 and both phosphoric esters. Additionally, ^13^C NMR analysis further revealed discernible chemical shift changes in the O‐*C*H_2_‐*C*H_2_‐O unit of the crown ether and the P‐O‐*C*H_2_‐ carbon signals of the phosphoric esters in the EMs (Figures S3–S6). FT‐IR spectroscopy corroborated these findings, showing clear shifts or bandwidth changes in the characteristic CH_2_‐O‐CH_2_ band of DtBuCH18C6 and the P = O group of HDEHP/EHEHPA (Figures S7–S10). Collectively, these results demonstrate that the successful formation of the DtBuCH18C6‐HDEHP and DtBuCH18C6‐EHEHPA EM systems is primarily attributed to extensive hydrogen‐bonding interactions between the ether oxygen atom of the crown ether and the P(= O)‐OH groups of the phosphoric esters. This understanding enables the rational construction of a novel class of functional materials.

### 2.2 Efficient Metal Ions Capture

**FIGURE 2:**
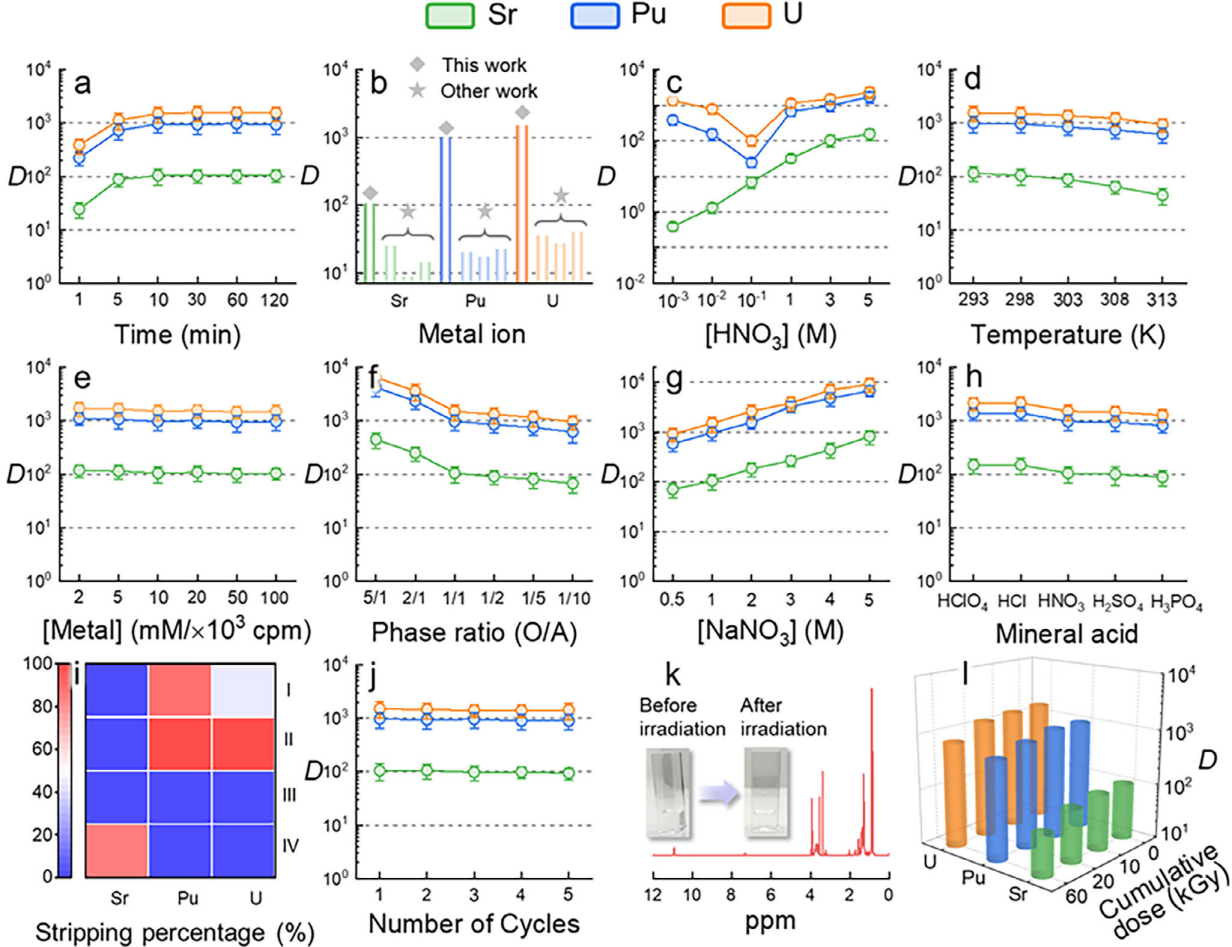
Metal ions recovery by DtBuCH18C6‐HDEHP EM. Effects of (a) contact time (1–120 min), (c) HNO_3_ concentration (10^−3^–5 M), (d) temperature (293–313 K), (e) metal ion content (2–100 mM) or radioactivity (2–100 × 10^3^ cpm/mL), (f) phase ratio (5/1 to 1/10), (g) NaNO_3_ concentration (0.5–5 M), (h) mineral acid medium, and (l) irradiation dose (0–60 kGy) on the uptake of U, Pu, and Sr. (b) Comparison of recovery performance with representative conventional systems. (i) Stripping and (j) cyclic tests of EM. (k) ^1^H NMR spectrum and image of EM sample induced by cumulative γ‐irradiation up to a total dose of 60 kGy. Standard experimental conditions. Initial organic phase: DtBuCH18C6‐HDEHP EM (1/2). Initial aqueous phase: 10 mM U(VI), 10 × 10^3^ cpm/mL Pu(IV), 10 mM Sr(II) mixed with 1 M NaNO_3_ in 3 M HNO_3_. Mixing 10 min at 298.0 ± 0.1 K with a phase ratio (O/A) of 1/1. The applied stripping agents were (I) 0.6 M oxalic acid/H_2_O solution, (II) 1 M Na_2_CO_3_/H_2_O solution, (III) 5 M HNO_3_, and (IV) 10^−3^ M HNO_3_. The γ irradiation dose rate was 5000 Gy/h, with irradiation durations of 2, 4, and 12 h, resulting in a total cumulative irradiation dose of 10, 20, and 60 kGy.

Given the highly acidic nature of nuclear waste solutions (typically 2–3 M HNO_3_) [30, 31] and the successful preparation of multifunctional eutectic mixtures described earlier, we systematically evaluated the recovery of critical ions (U(VI), Pu(IV), and Sr(II)) from HNO_3_ medium through solvent extraction under multiple influencing factors. Four EMs, namely, DtBuCH18C6‐HDEHP EM with molar ratios of 1/2 and 1/8, and DtBuCH18C6‐EHEHPA EM with molar ratios of 1/2 and 1/8, were assessed and compared in terms of their capture efficiency. The results indicate that all four EMs exhibit broadly similar uptake trends (Figure 2, Figures S11–S13). For clarity, the DtBuCH18C6‐HDEHP EM (1/2) system is selected as a representative case for detailed discussion.

This integrated EM demonstrates exceptional metal‐capture kinetics, reaching equilibrium within 10 min (Figure 2a). Under standard experimental conditions (in the annotation of Figure 2), the material enables highly efficient and simultaneous removal of all target ions, with distribution ratios (*D*) as high as 1500 for U(VI), 970 for Pu(IV), and 104 for Sr(II). This corresponds to single‐stage recovery efficiencies exceeding 99.9%, 99.8%, and 99%, respectively, not only fulfilling our design objective but also surpassing the performance of several representative systems conventionally employed for the direct treatment of spent nuclear fuel, exhibiting an enhancement by factors ranging from severalfold to several tens of times (Figure 2b) [14, 32, 33, 34, 35, 36, 37]. Comparative analysis reveals that a higher HDEHP proportion in the DtBuCH18C6‐HDEHP EM moderately reduces the recovery efficiency for Pu(IV) and Sr(II). A similar trend is observed when HDEHP is replaced by EHEHPA (Figure 2, Figures S11–S13), underscoring the significant influence of both EM composition and component ratio on ion capture behavior. Furthermore, the uptake of U(VI) and Pu(IV) initially decreases and then increases with rising acidity, whereas Sr(II) recovery exhibits a consistently positive correlation with acid concentration (Figure 2c). This divergence arises from the distinct mechanisms governed by the HBD and HBA molecules within the EM composite. As an acidic extractant, HDEHP experiences suppressed dissociation of its phosphate group under moderately increasing acidity, thereby impairing its affinity toward U(VI) and Pu(IV). However, in molar‐range acidity, the P(= O)‐OH group no longer dissociates, effectively converting HDEHP into a neutral alkylphosphine oxide‐like species with non‐dissociating “pseudo‐alkyl” chains. Under such conditions, high concentrations of NO_3_^−^ as counterion facilitate metal ion uptake [38]. Similarly, DtBuCH18C6, as a neutral extractant, benefits from high acid media where elevated anion concentrations enhance Sr(II) capture [9, 14].

To assess the system's applicability under realistic and challenging conditions, such as high heat release from radiolysis and high salinity, we systematically evaluated the EM's performance across various parameters. Elevated temperatures led to a decline in recovery efficiency for all ions (Figure 2d), indicating an exothermic metal‐ligand complexation process, consistent with reported thermodynamic behavior of crown ether‐ and phosphonate‐based systems. Variations in initial metal ion concentration/radioactivity showed negligible effects on ion‐uptake performance (Figure 2e). Considering the need for flexible phase flow ratio adjustment in industrial‐scale separations, we further examined extraction across a wide range of organic/aqueous (O/A) phase ratios. Although reducing the O/A ratio from 5/1 to 1/1 resulted in decreased efficiency, further reduction had minimal impact (Figure 2f). Even at an O/A ratio as low as 1/10, *D* values remained as high as 960 for U(VI), 620 for Pu(IV), and 66 for Sr(II), demonstrating robust capture across diverse flow conditions. Increasing ionic strength markedly enhanced the uptake of all target ions (Figure 2g), attributable to the salting‐out effect in high‐salt environments [21, 39]. The acid medium also influenced recovery performance. HCl and HClO_4_ systems slightly outperformed those based on H_2_SO_4_, HNO_3_, and H_3_PO_4_, likely due to the stronger complexation ability of SO_4_^2−^, NO_3_^−^, and PO_4_^3−^ with metal ions, which enhances masking effects (Figure 2h). Under all tested conditions, the EM maintained exceptionally high distribution ratios (>1000 for U(VI), >500 for Pu(IV), and >50 for Sr(II)), highlighting its broad adaptability across wide ranges of acidity, temperature, metal content, phase ratio, salinity, and mineral acid media.

To enable recyclability, we investigated the stripping behavior of metal‐loaded EM. Owing to the distinct chemical properties of U(VI), Pu(IV), and Sr(II), their responses to different stripping agents varied significantly. Overall, 10^−3^ M HNO_3_ efficiently stripped Sr(II) with >85% single‐stage efficiency, while 1 M Na_2_CO_3_ aqueous solution afforded near‐quantitative recovery of both U(VI) and Pu(IV), with efficiencies reaching 98% and 97%, respectively (Figure 2i). The regenerated EM retained excellent nuclide uptake capacity over five consecutive extraction‐stripping cycles (Figure 2j). Given the inherently high radioactivity of nuclear waste, we further evaluated the radiolytic stability of the EM and its post‐irradiation performance. Clearly, irradiated samples exhibited no discernible macroscopic alterations (Figure 2k). Semi‐quantitative integration of characteristic NMR signals (Figure 2k) indicated that radiolysis products accounted for ≤3% of the total composition, suggesting that the majority of the EM remained intact. Extraction tests performed on samples subjected to varying cumulative irradiation doses revealed only a moderate decline in performance with increasing dose, confirming that the EM retains effective capture capability for U(VI), Pu(IV), and Sr(II) even after intense irradiation (Figure 2l). These findings collectively underscore the significant potential of this kind of integrated EM system for the uptake and recovery of critical metal ions from nuclear waste under extreme and realistic environmental conditions.

### 2.3 Mechanistic Insights

**FIGURE 3:**
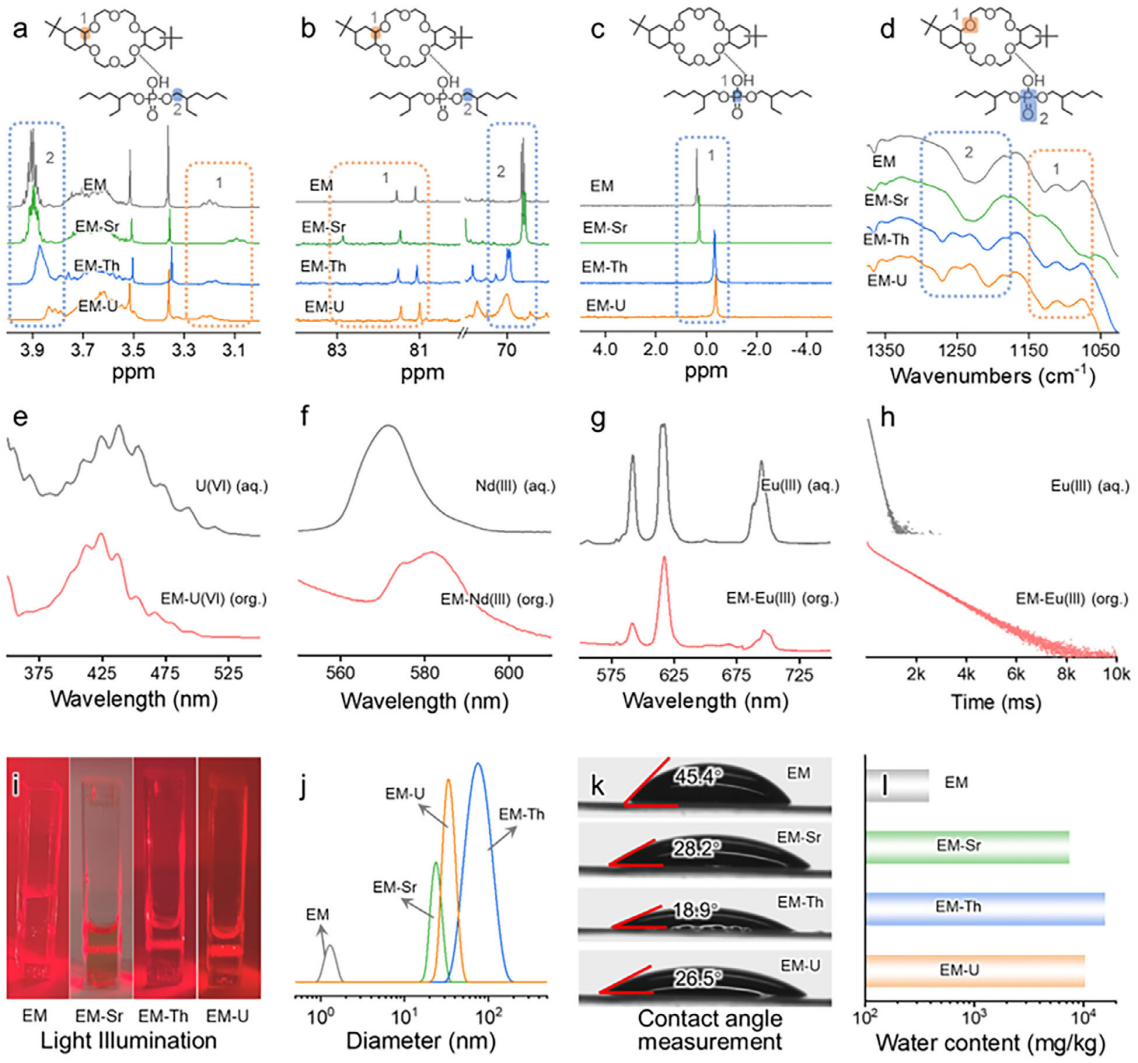
Analysis of the interaction between DtBuCH18C6‐HDEHP EM and metal ions. Comparison of “free” and metal‐incorporated DtBuCH18C6‐HDEHP EM through (a) ^1^H NMR, (b) ^13^C NMR, (c) ^31^P NMR, and (d) FT‐IR spectra. Absorption spectra of (e) U and (f) Nd‐incorporated DtBuCH18C6‐HDEHP EM. (g) Luminescence emission spectra and (h) representative decay patterns of Eu‐incorporated DtBuCH18C6‐HDEHP EM. (i) Graphs, (j) particle size, (k) contact angle, and (l) water content of the DtBuCH18C6‐HDEHP EM phase before and after metal ion‐incorporation. Experimental conditions. Organic phase: (a–l) DtBuCH18C6‐HDEHP EM (1/2). Aqueous phase: (a–d) 10 mM U(VI), 10 mM Th(IV), 10 mM Sr(II), (e) 10 mM U(VI), (f) 10 mM Nd(III), (g,h) 10 mM Eu(III) and (i–l) a mixture of 10 mM each of U(VI), Th(IV), and Sr(II) in 3.0 M HNO_3_. The metal ions were incorporated into the organic solution through solvent extraction.

The exceptional performance of the constructed multifunctional EMs toward key components in nuclear waste has motivated us to systematically investigate the underlying interaction mechanisms. Given the experimental constraints of handling radionuclides, and to ensure a comprehensive and reliable mechanistic analysis, we selected Th(IV), Nd(III), and Eu(III) as surrogate ions. These f‐block elements exhibit chemical behaviors analogous to Pu(IV) but are characterized by low or no radioactivity [40]. In addition, considering that the afore‐experiments of metal uptake have revealed the similarity in chemical behaviors between the DtBuCH18C6‐HDEHP EM and DtBuCH18C6‐EHEHPA EM, and given that HDEHP and EHEHPA inherently belong to the same class of molecular types, the optimal performing DtBuCH18C6‐HDEHP EM (1/2) system was selected for detailed investigation (Figure 3).

^1^H, ^13^C, and ^31^P NMR analyses revealed that the incorporation of Sr(II) induced noticeable chemical shift changes in the H and C atoms adjacent to the ether O atoms in DtBuCH18C6 (Figure 3a,b). In contrast, the loading of Th(IV) and U(VI) primarily perturbed the H/C atoms near the P(= O)‐O‐ group of HDEHP, along with a detectable shift in the P signal (Figure 3a–c). These observations suggest that within the DtBuCH18C6‐HDEHP EM, DtBuCH18C6 is predominantly responsible for recognizing and capturing Sr(II), whereas HDEHP plays a more direct role in coordinating actinide ions. FT‐IR analysis further confirmed these interactions, showing marked changes in the characteristic bands of both the ether O and P = O functional groups upon metal uptake (Figure 3d). Subsequent in situ spectroscopic analysis revealed notable alterations in the spectral profiles of metal ions upon their incorporation into the EM phase. Specifically, the absorption spectra of U(VI) and Nd(III) (Figure 3e,f) and the luminescence emission spectrum of Eu(III) (Figure 3g) were significantly modified, indicating substantial changes in their coordination environments. Notably, the fluorescence decay lifetime (*τ*) of the Eu(III) complex, with components at 1048 µs (88%), 397 µs (9%), and 244 µs (3%), was considerably longer than that in the aqueous phase (130 µs, Figure 3h). Using Equation (1) [41, 42], the number of water molecules in the first coordination sphere of Eu(III) was determined to be 7–8 in aqueous solution and 0–4 in the complex, indicating that water molecules in the inner coordination sphere were partially displaced by organic constituents of the EM during extraction—one of the key mechanisms facilitating efficient metal capture.

(1) $$N\;\left(H_{2}O\right)=\frac{1.05}{\tau }\;-0.44$$

Furthermore, a distinct Tyndall effect was observed in the organic phase after recovering a mixed solution containing U(VI), Th(IV), and Sr(II) (Figure 3i), suggesting the presence of supramolecular aggregates beyond simple coordination complexes. To verify this hypothesis, the EM‐metal systems were further analyzed by dynamic light scattering (DLS), which revealed the existence of nanostructures with a size distribution of 10–200 nm (Figure 3j). Given the amphiphilic nature of phosphoric esters, featuring hydrophilic P(= O)‐OH groups and hydrophobic ‐(CH_2_)_n_CH_3_ chains, we propose that the hydrated metal ions promote the self‐assembly of HDEHP or EHEHPA into reverse micelle‐like supramolecular entities, a phenomenon previously documented in conventional solvent extraction systems [43, 44, 45, 46]. Contact angle measurements further indicated a gradual decrease in the interfacial contact angle of the EM with increasing metal uptake (Figure 3k), reflecting enhanced surface tension and internal hydration. Water content analysis corroborated these findings, showing a significant rise in hydration levels after metal recovery, which became more pronounced for the ion with higher charge density (Figure 3l). These results support the hypothesis that metal capture in this EM system proceeds through the formation of reverse micelle‐type supramolecular assemblies.

**FIGURE 4:**
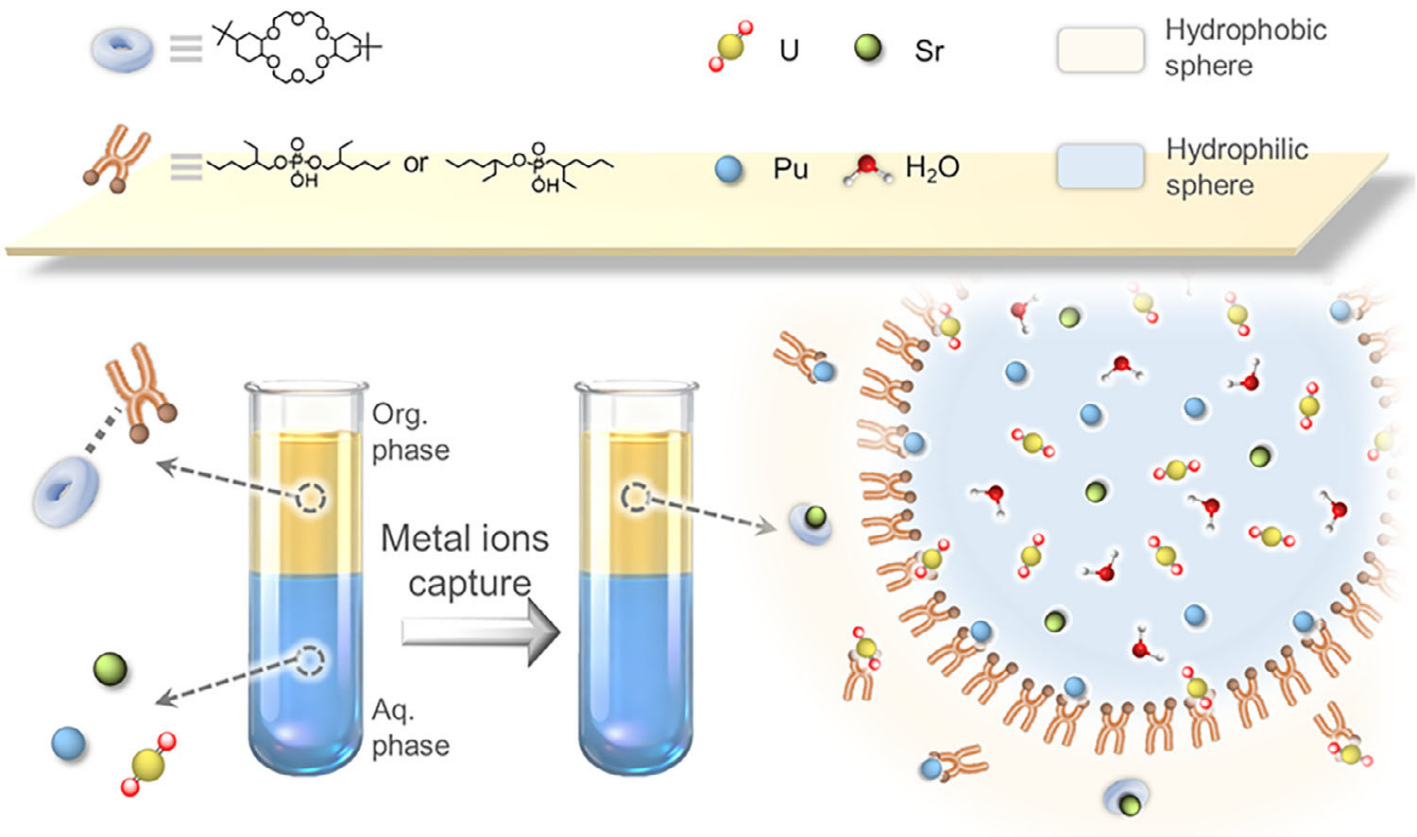
Schematic diagram of the proposed mechanism for metal ions capture by the eutectic mixtures.

Based on the above findings, we propose a mechanistic model for the deep removal of U, Pu, and Sr by the integrated EM material, as illustrated in Figure 4. On one hand, the crown ether and phosphoric esters within the EM system targeted capture Sr and actinide ions, respectively, through differentiated coordination: the crown ether preferentially complexes Sr(II), while the phosphonate species primarily extract actinide ions. On the other hand, the EM contains a substantial excess of phosphoric ester molecules from a stoichiometric perspective. Owing to the amphiphilic nature, featuring both hydrophilic headgroups and hydrophobic tails, these molecules facilitate the spontaneous self‐assembly of reverse micelle‐like supramolecular structures upon contact with metal ions. This unique architecture enables highly efficient sequestration and stabilization of the target metal ions via a synergistic “coordination‐encapsulation” mechanism. The formation of such reverse micellar compartments represents an approach that transcends the limitations of conventional systems that rely solely on individual coordination interactions, thereby enhancing, at the molecular assembly level, the overall recovery performance and leading to superior metal ion capture compared to traditional methods. It is the cooperative interplay of these two mechanisms that empowers the system to achieve highly efficient and simultaneous recovery of multiple target elements in multi‐component and chemically complex environments.

## 3 Conclusions

In this work, we successfully designed and constructed a novel class of multifunctional EMs based on DtBuCH18C6 and HDEHP/EHEHPA, which serve as an integrated and diluent‐free platform for the highly efficient, simultaneous recovery of strategic elements (U, Pu, Sr) from nuclear waste. The material self‐assembles via hydrogen bonding and exhibits exceptional performance. Mechanistic studies reveal that this remarkable efficacy originates from the differentiated coordination behaviors of the crown ether and phosphonate units, synergistically enhanced by a unique “coordination‐encapsulation” mechanism within a reverse micelle‐like supramolecular architecture. More importantly, the developed EMs demonstrate robust radiation resistance, strong adaptability to complex chemical environments, and satisfactory recyclability, highlighting their great promise for practical application under harsh conditions. Consequently, this work not only delivers a high‐performance material for nuclear waste treatment and sustainable nuclear fuel cycles but also provides fundamental insights into the role of molecular assembly in multi‐component separation, thereby establishing a solid theoretical and experimental foundation for the development of advanced separation materials.

## 4 Experimental Section

### 4.1 Chemicals and Materials

Stock solutions of Th(IV)‐232, U(VI)‐238, and Pu(IV)‐239 were supplied by the Institute of Nuclear and New Energy Technology (INET) at Tsinghua University. Sr(NO_3_)_2_, Nd(NO_3_)_3_, Eu(NO_3_)_3_, Na_2_CO_3_, oxalic acid, NaNO_3_, HNO_3_, HClO_4_, HCl, H_2_SO_4_, H_3_PO_4_ were procured from Aladdin Reagent Co., Ltd. HDEHP and EHEHPA were obtained from Macklin Reagent Company. DtBuCH18C6 was synthesized in our laboratory according to a reported procedure. All experiments utilized Milli‐Q purified water. Unless otherwise stated, all chemicals were of analytical grade or higher. *Caution: Pu(IV)‐239 is a highly radioactive species and poses significant health risks. Consequently, all experiments involving this and other radionuclides were performed exclusively within gloveboxes specifically designed for the safe manipulation of radioactive materials*.

### 4.2 Preparation of EMs

EMs were prepared by combining DtBuCH18C6 with the respective organophosphorus molecule (HDEHP or EHEHPA) at molar ratios of 1:2 or 1:8 (crown ether:phosphoric ester) in sealed glass vials. The mixtures were subjected to ultrasonication for 0.5 h until a clear, homogeneous liquid formed. The resulting mixtures were subsequently allowed to stand for 24 h to evaluate their stability. No phase separation or precipitation was observed, confirming the successful formation and inherent stability of the target EMs.

### 4.3 Measurements and Characterizations

NMR spectra were acquired on a Bruker Avance III 600 MHz spectrometer (Bruker, Inc.). Fourier‐Transform Infrared (FT‐IR) spectra were recorded using a Nicolet iZ10 spectrophotometer (Thermo Fisher Scientific). The concentration of Pu was quantified using an ultra‐low background liquid scintillation spectrometer (Quantulus 1220, PerkinElmer). Concentrations of U and Sr were determined by Inductively Coupled Plasma Optical Emission Spectrometry (ICP‐OES, Agilent, Inc.). UV–Vis–NIR absorption spectra were collected on a Cary 6000i spectrophotometer (Agilent, Inc.). Luminescence emission spectra were measured with an Edinburgh FLS‐1000 spectrophotometer equipped with a 450 W ozone‐free xenon arc lamp. DLS measurements were performed on a Malvern Nano ZS90 instrument at 298.0 ± 0.1 K with a fixed scattering angle of 90°. Contact angles were determined using an optical contact angle measurement and contour analysis system (OCAH 200, Dataphysics). Water content in the EMs was assessed via Karl Fischer titration (851 KF Titrando, Metrohm) employing the coulometric method with solvent‐based sample introduction. Irradiation stability tests were performed using a Cobalt‐60 (^60^Co) source at Tsinghua University (activity: 1.3 × 10^15^ Bq). The absorbed dose rate was modulated by adjusting the distance between the sample and the radiation source.

### 4.4 Solvent Extraction and Back Extraction

Solvent extraction experiments were carried out using the synthesized eutectic mixtures (DtBuCH18C6‐HDEHP EM or DtBuCH18C6‐EHEHPA EM) as the organic phase. The aqueous phase consisted of HNO_3_ solutions containing a single metal ion (U(VI), Pu(IV), or Sr(II)) at a specified concentration, adjusted with NaNO_3_ to maintain the desired ionic strength. In a standard procedure, the organic and aqueous phases were combined in stoppered glass tubes with a specific phase ratio and agitated for a predetermined time at a controlled temperature. Phase separation was achieved by centrifugation.

For Pu analysis, 100 µL aliquots from both phases were transferred into 10 mL plastic scintillation vials, mixed with 3 mL of Hisafe 3 scintillation cocktail, and the radioactivity was measured using the ultra‐low background liquid scintillation spectrometer. For U and Sr analysis, aqueous phase samples before and after extraction were collected, diluted to an appropriate fold, and analyzed by ICP‐OES. The distribution ratio (*D*) was calculated as *D* = *C*_org._/*C*_aq._, where *C*_org._ and *C*_aq._ represent the equilibrium concentrations of the target metal ion in the organic and aqueous phases, respectively. The extraction efficiency (*E*) can be defined as the quotient of the concentration of metal ions extracted into the organic phase (*C*_org._) to the total initial concentration of metal ions in the aqueous phase (*C*_aq._ + *C*_org._). It can be calculated using the equation: *E* = *C*_org._/(*C*_aq._ + *C*_org._) × 100%. The relationship between *E* and *D* is given by: *E* = *D*/(*D* + 1) × 100%. Accordingly, distribution ratios of *D* = 9, 99, and 999 correspond to extraction efficiencies of *E* = 90%, 99%, and 99.9%, respectively.

For back‐extraction (stripping) experiments, 1.0 mL of the metal‐loaded EM was contacted with 1.0 mL of an appropriate stripping solution in a stoppered glass tube and mixed for 10 min. After phase separation by centrifugation, samples were analyzed according to the protocols described above. The stripping efficiency (%) was calculated as the percentage of the cation recovered into the aqueous phase relative to its initial mass loaded in the organic phase. All extraction (except irradiation performance test) and back‐extraction experiments were performed in triplicate to ensure the reproducibility and reliability of the data.

## Conflicts of Interest

The authors declare no conflicts of interest.

## Supporting information




**Supporting File**: advs75174‐sup‐0001‐SuppMat.docx.

## Data Availability

The data that support the findings of this study are available from the corresponding author upon reasonable request.
