# Supplementary material for: Multifunctional Eutectic Mixtures Enable Integrated Recovery of U, Pu, and Sr From Nuclear Waste
Source: Adv Sci (Weinh). 2026 Apr 3;13(36):e75174. doi: 10.1002/advs.75174 (PMC13317632; doi:10.1002/advs.75174)
Supplement: Supplementary file 1 — Supporting File: advs75174‐sup‐0001‐SuppMat.docx. [file ADVS-13-e75174-s001.docx]

Supporting Information

Multifunctional Eutectic Mixtures Enable Integrated Recovery of U, Pu, and Sr from Nuclear Waste

*Yifan Wang, Yanjun Lv, Qilong Tang, Huaixin Hao, Jianfeng Jia, Gang Ye, Jing Chen, Chao Xu^*^, Zhipeng Wang^*^*

Dr. Yifan Wang, Dr. Yanjun Lv, Dr. Qilong Tang, Dr. Huaixin Hao, Dr. Jianfeng Jia, Prof. Gang Ye, Prof. Jing Chen, Prof. Chao Xu, Prof. Zhipeng Wang

Institute of Nuclear and New Energy Technology, Tsinghua University, Beijing 100084, China

Dr.Yanjun Lv

College of Sciences, Northeastern University, Shenyang 110004, China

*Corresponding author E-mails: xuchao@tsinghua.edu.cn, wangzhipeng@mail.tsinghua.edu.cn


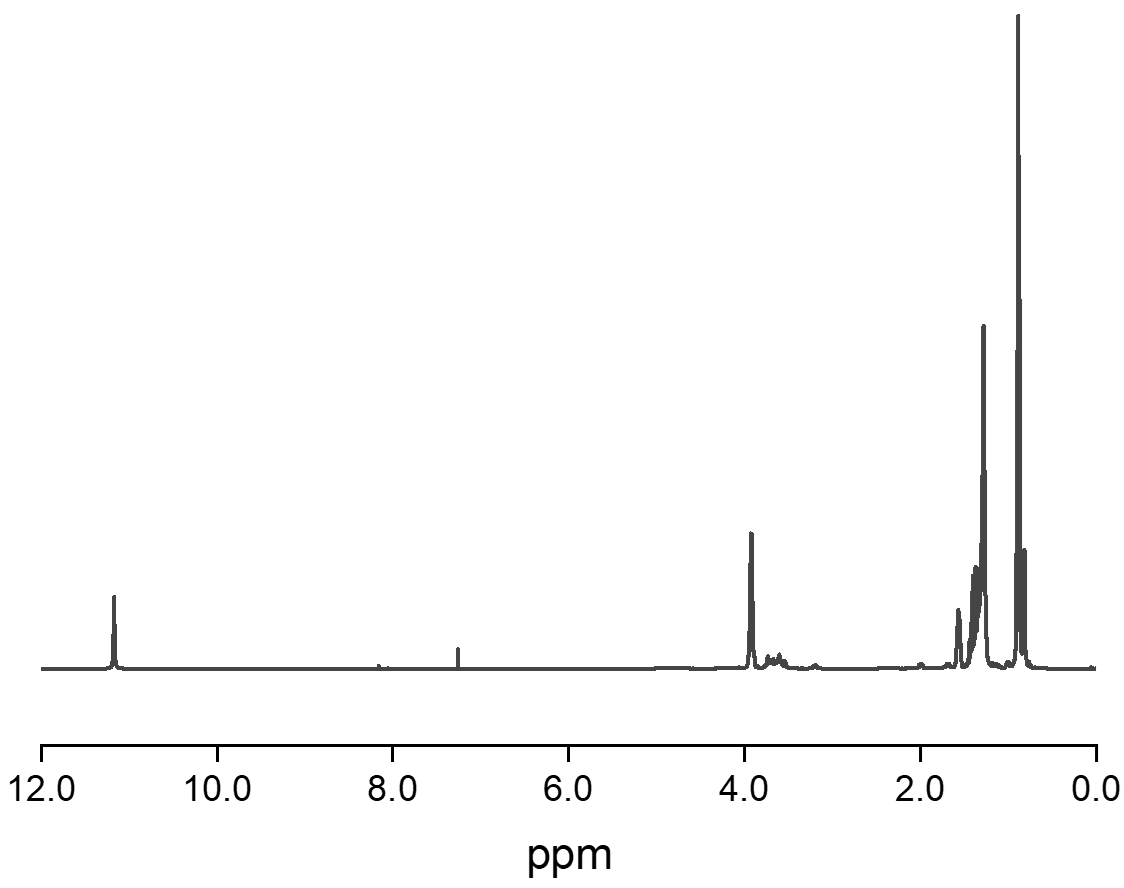


**Figure S1.** ^1^H NMR spectra of DtBuCH18C6-HDEHP EM (molar ratio 1/8).


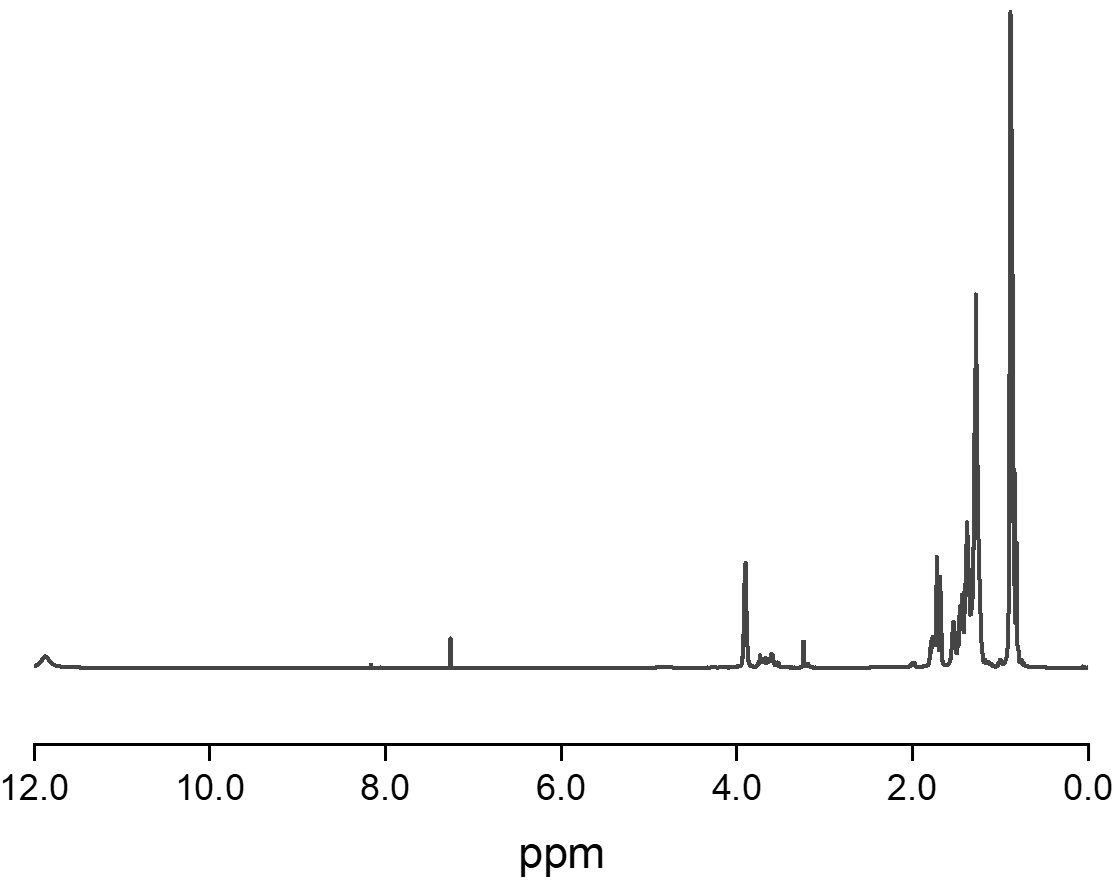


**Figure S2.** ^1^H NMR spectra of DtBuCH18C6-EHEHPA EM (molar ratio 1/8).


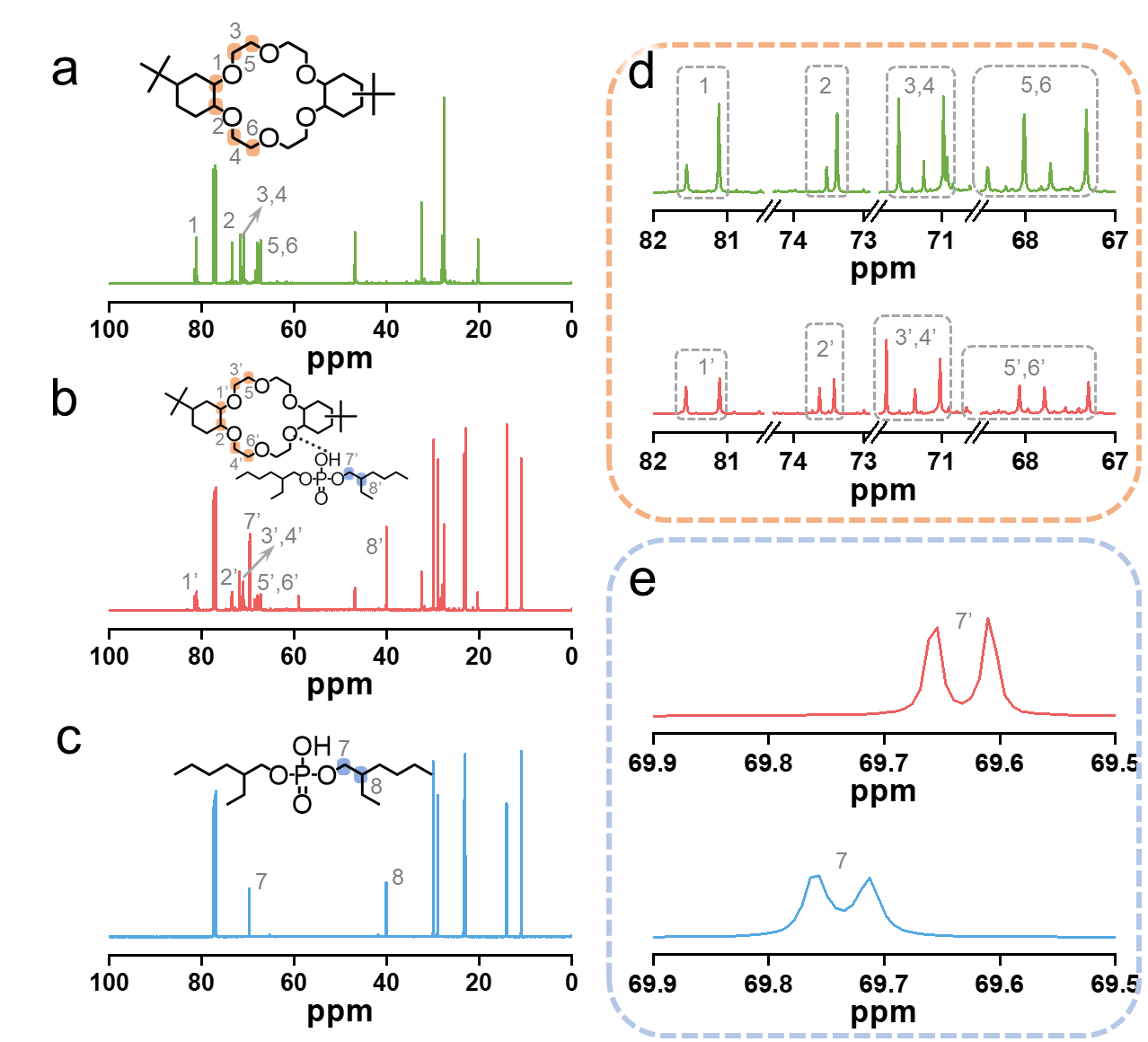


**Figure S3.** ^13^C NMR spectra of (a) DtBuCH18C6, (b) DtBuCH18C6-HDEHP EM (molar ratio 1/2), and (c) HDEHP. (d,e) Expanded spectral regions for comparative analysis.


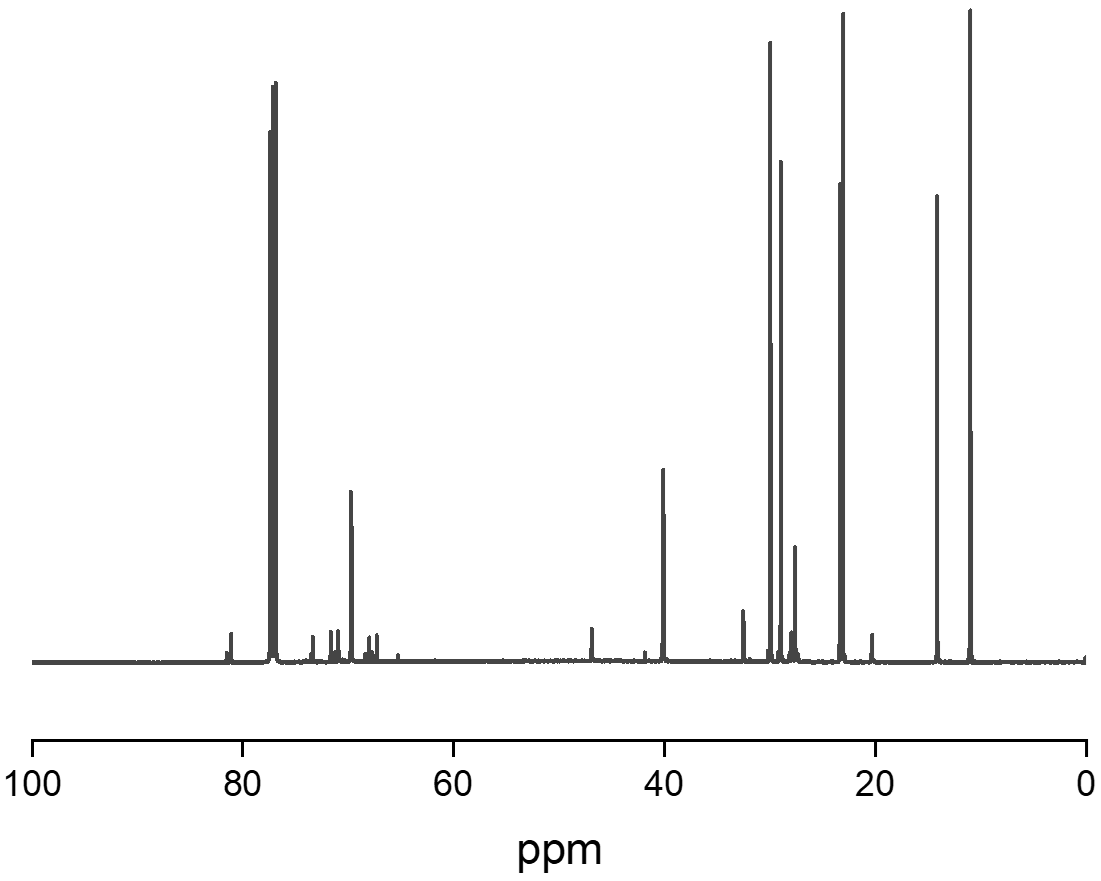


**Figure S4.** ^13^C NMR spectra of DtBuCH18C6-HDEHP EM (molar ratio 1/8).


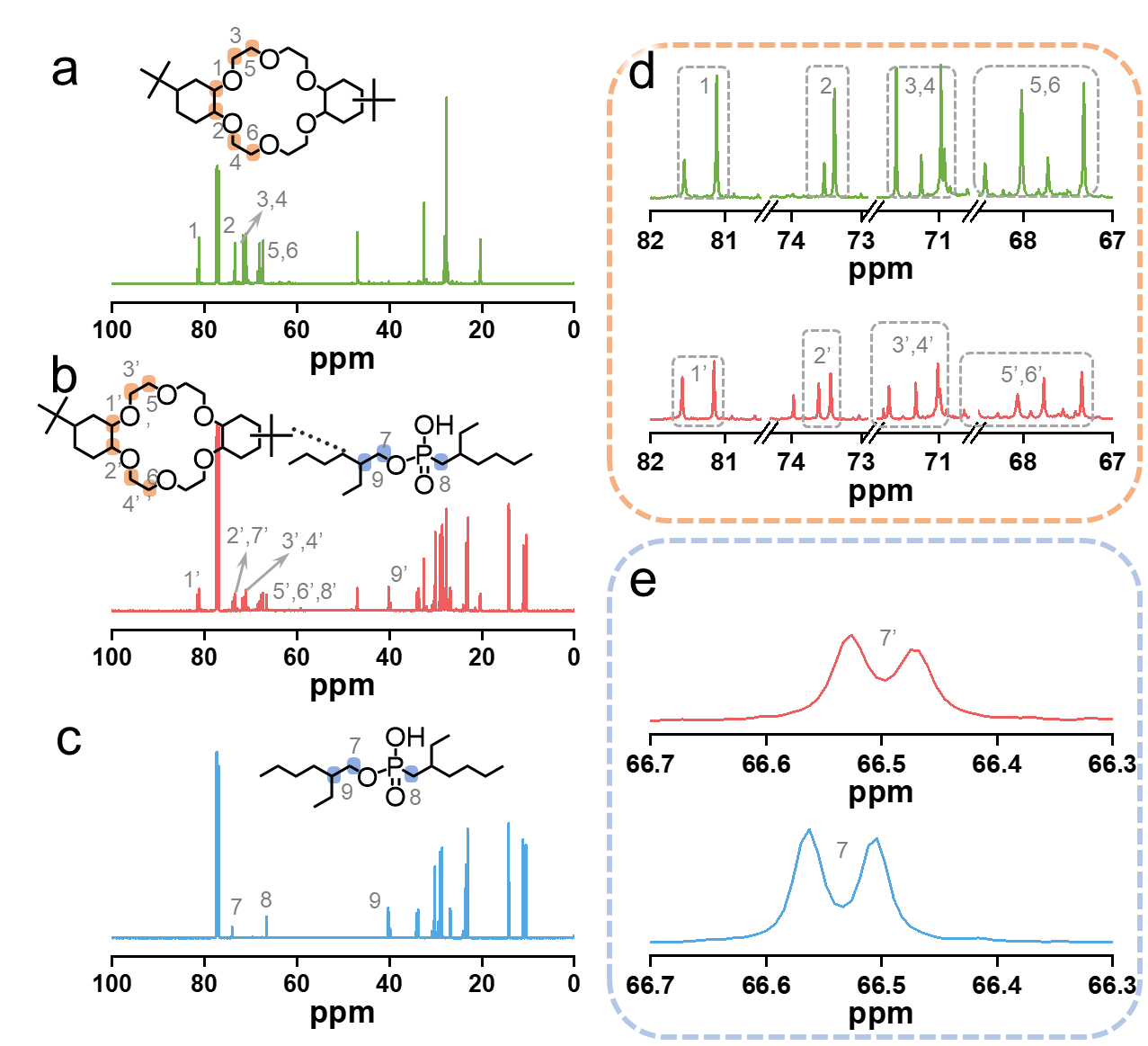


**Figure S5.** ^13^C NMR spectra of (a) DtBuCH18C6, (b) DtBuCH18C6-EHEHPA EM (molar ratio 1/2), and (c) EHEHPA. (d,e) Expanded spectral regions for comparative analysis.


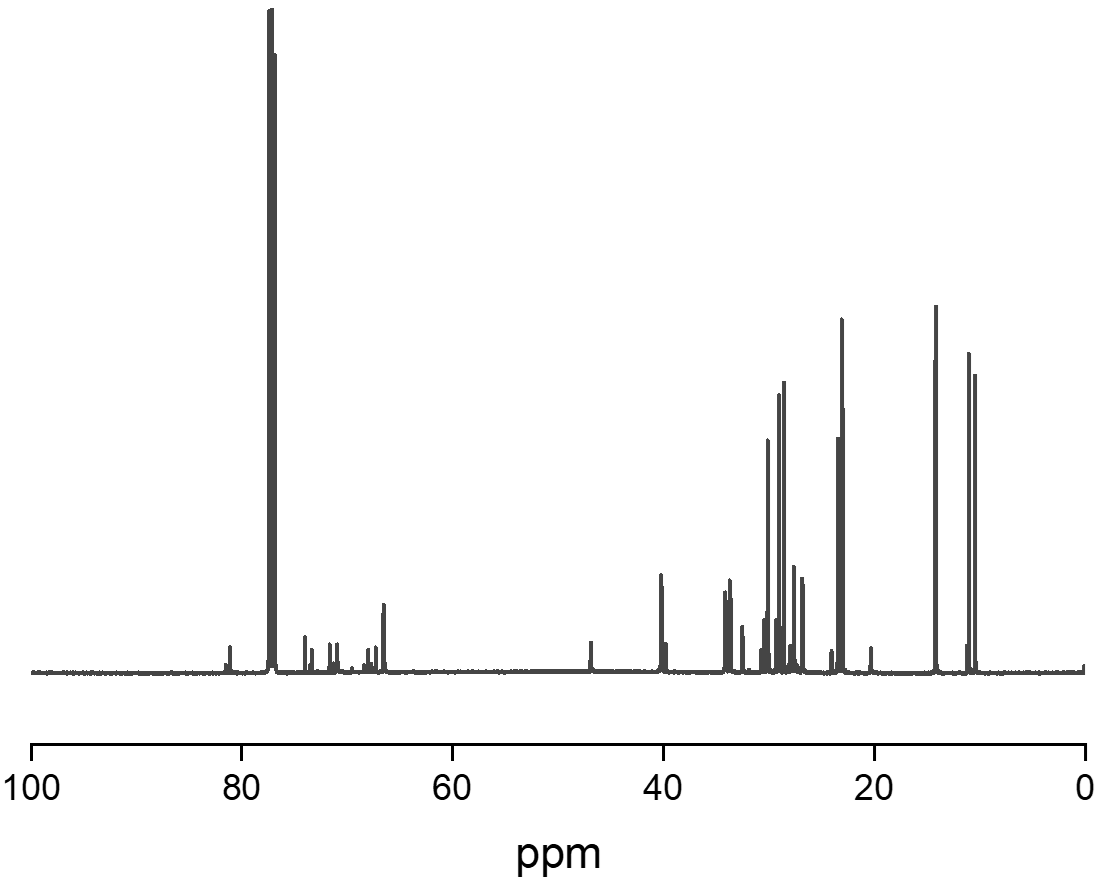


**Figure S6.** ^13^C NMR spectra of DtBuCH18C6-EHEHPA EM (molar ratio 1/8).


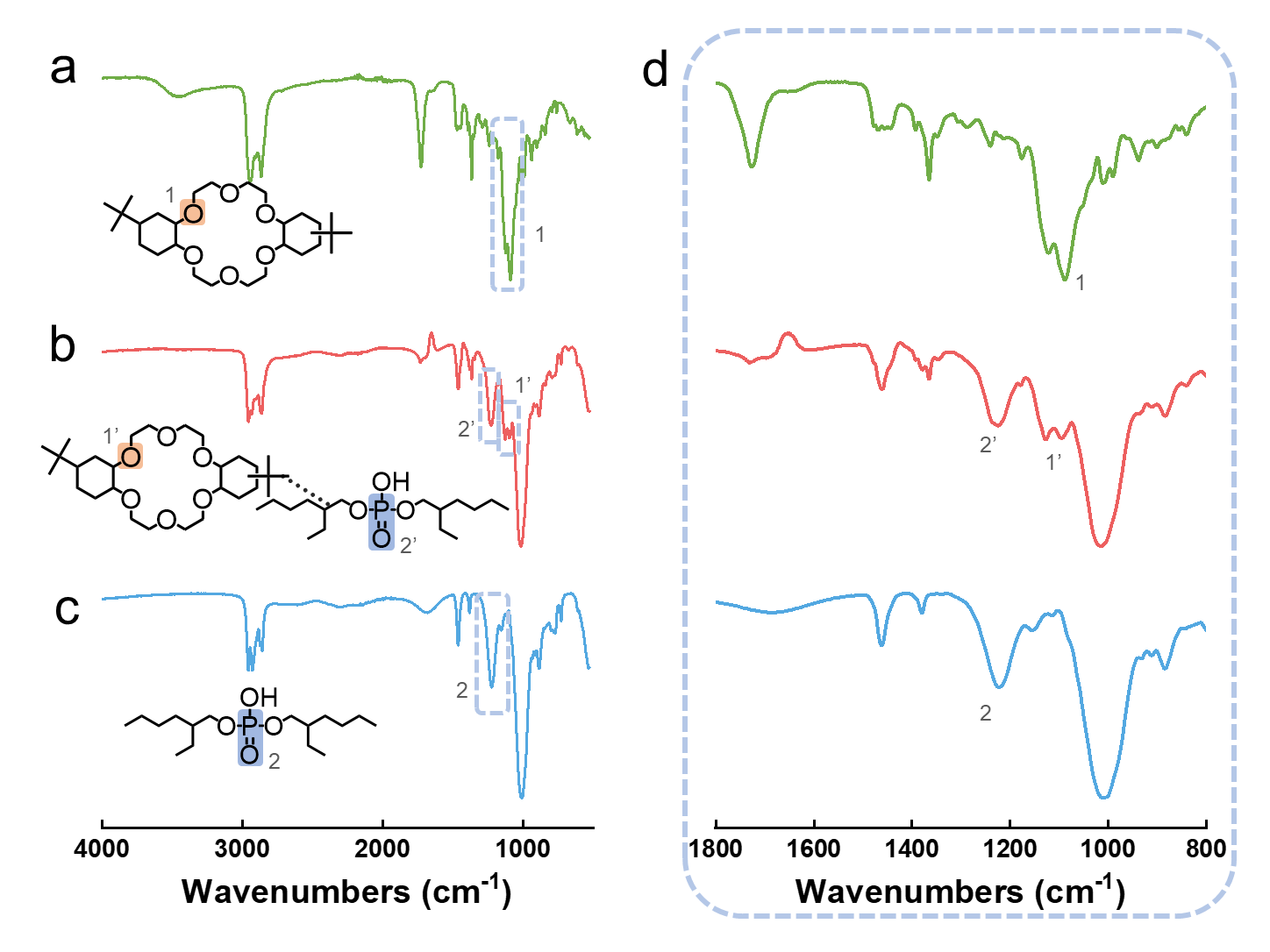


**Figure S7.** FT-IR spectra of (a) DtBuCH18C6, (b) DtBuCH18C6-HDEHP EM (molar ratio 1/2), and (c) HDEHP. (d) Expanded spectral regions for comparative analysis.


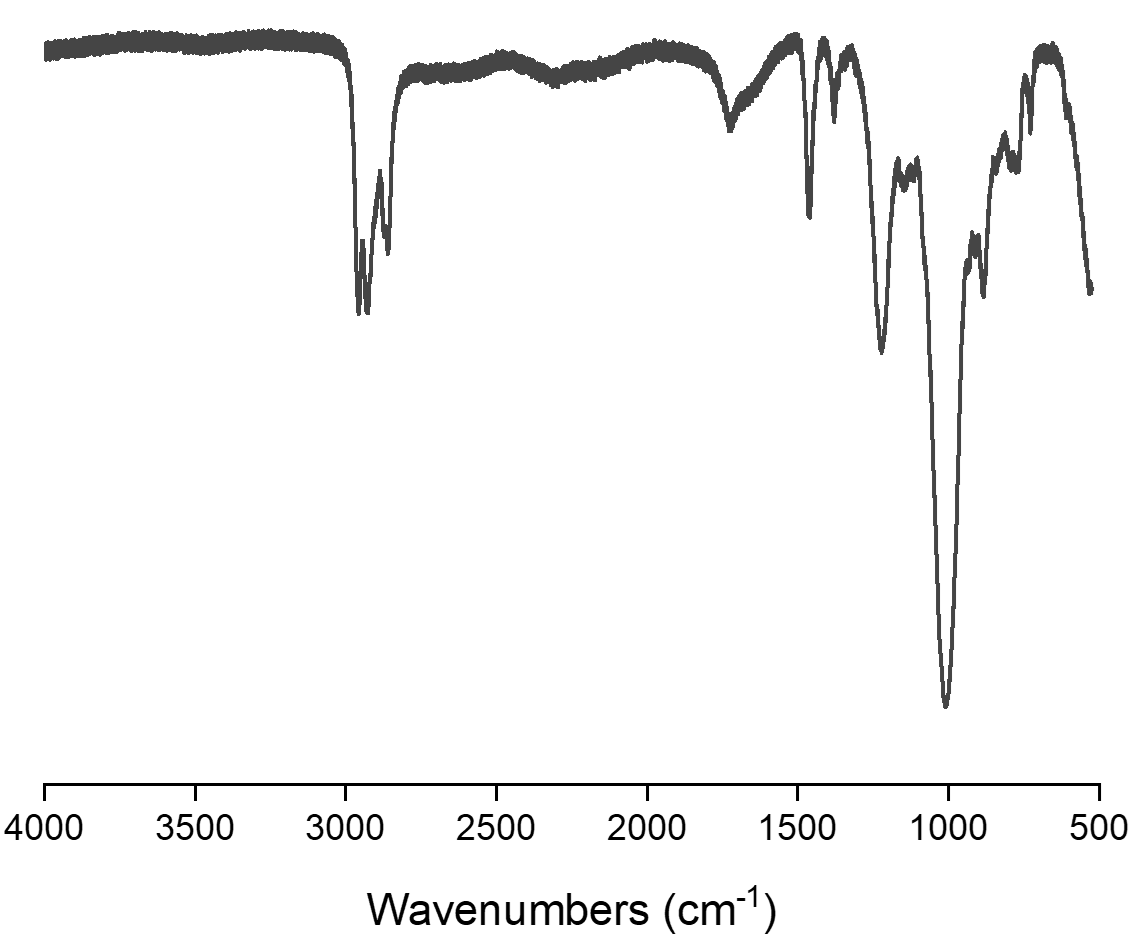


**Figure S8.** FT-IR spectra of DtBuCH18C6-HDEHP EM (molar ratio 1/8).


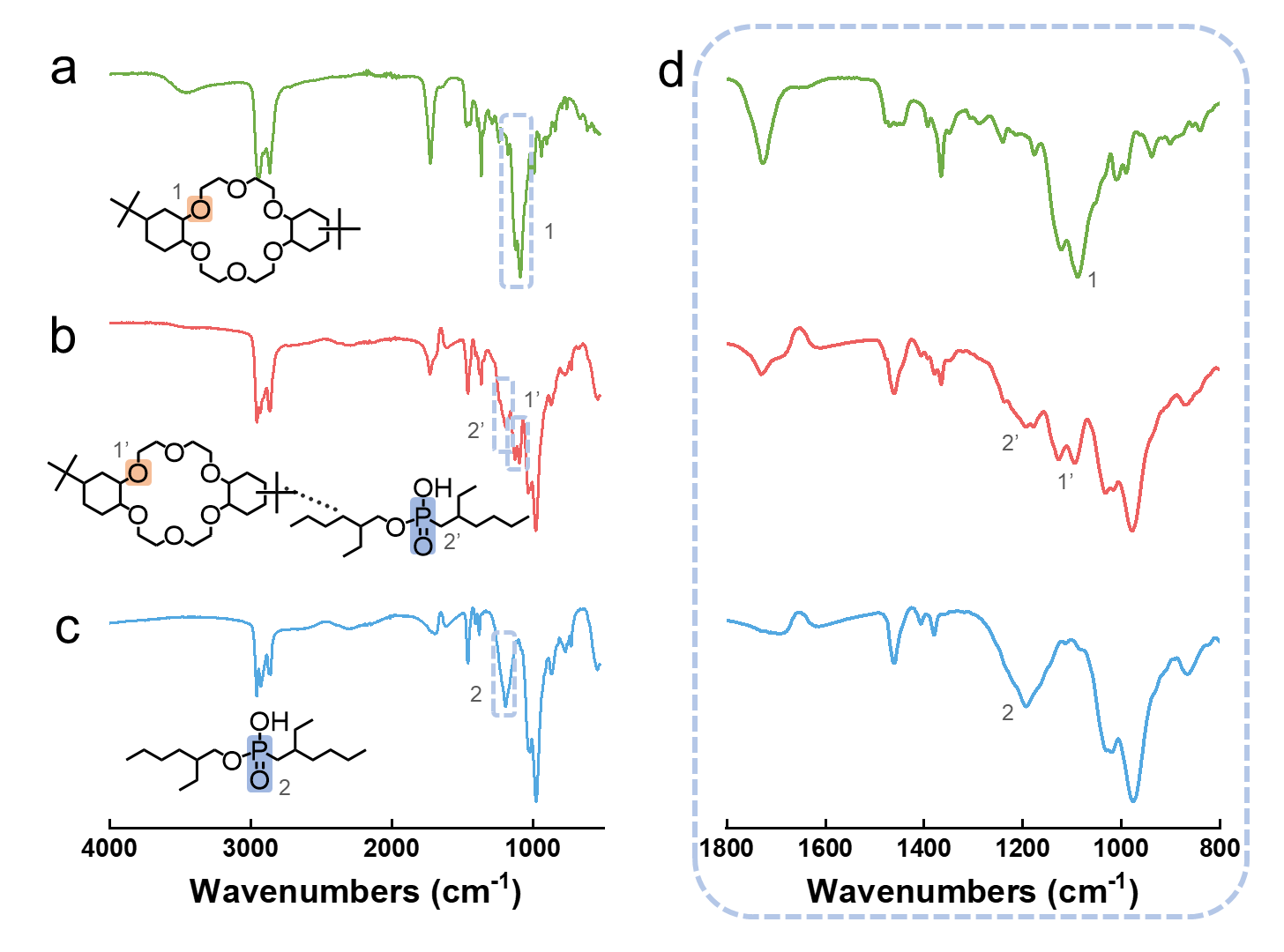


**Figure S9.** FT-IR spectra of (a) DtBuCH18C6, (b) DtBuCH18C6-EHEHPA EM (molar ratio 1/2), and (c) EHEHPA. (d) Expanded spectral regions for comparative analysis.


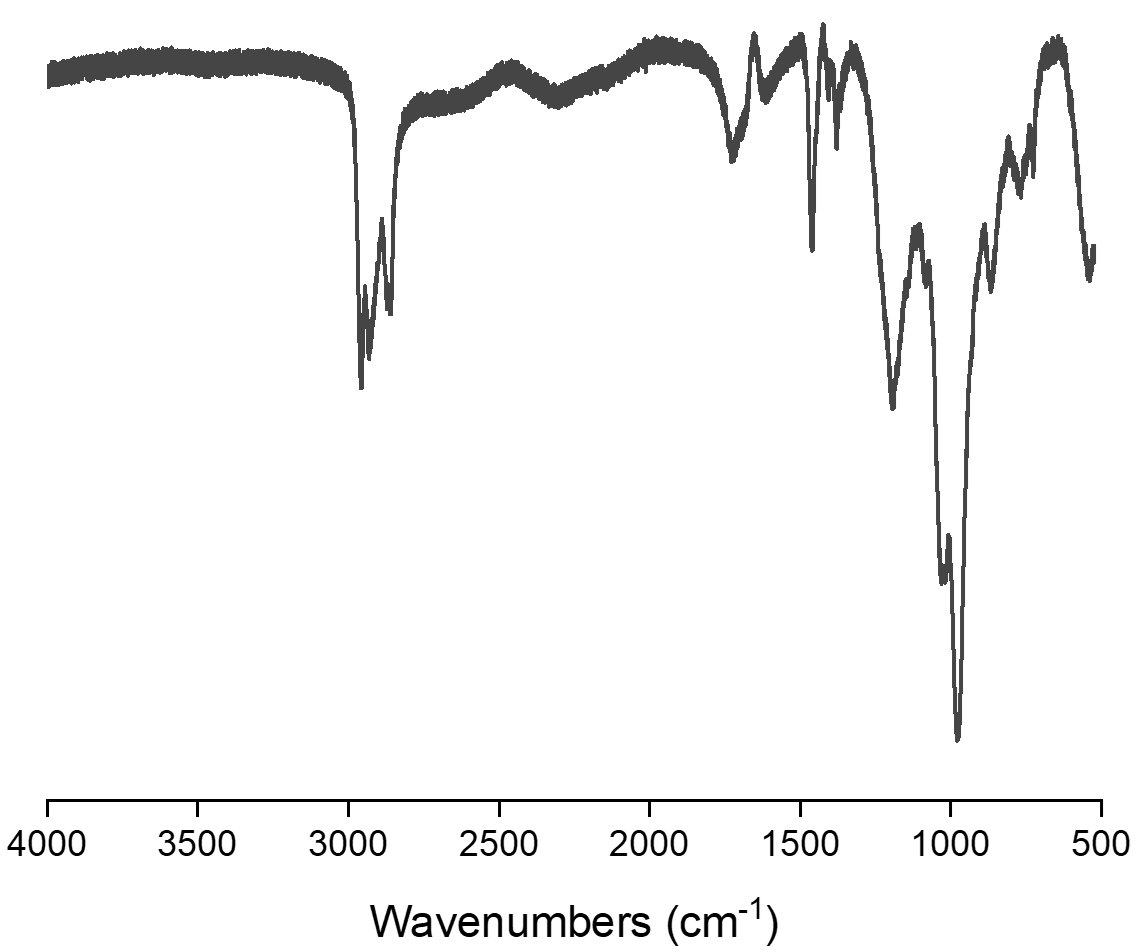


**Figure S10.** FT-IR spectra of DtBuCH18C6-EHEHPA EM (molar ratio 1/8).


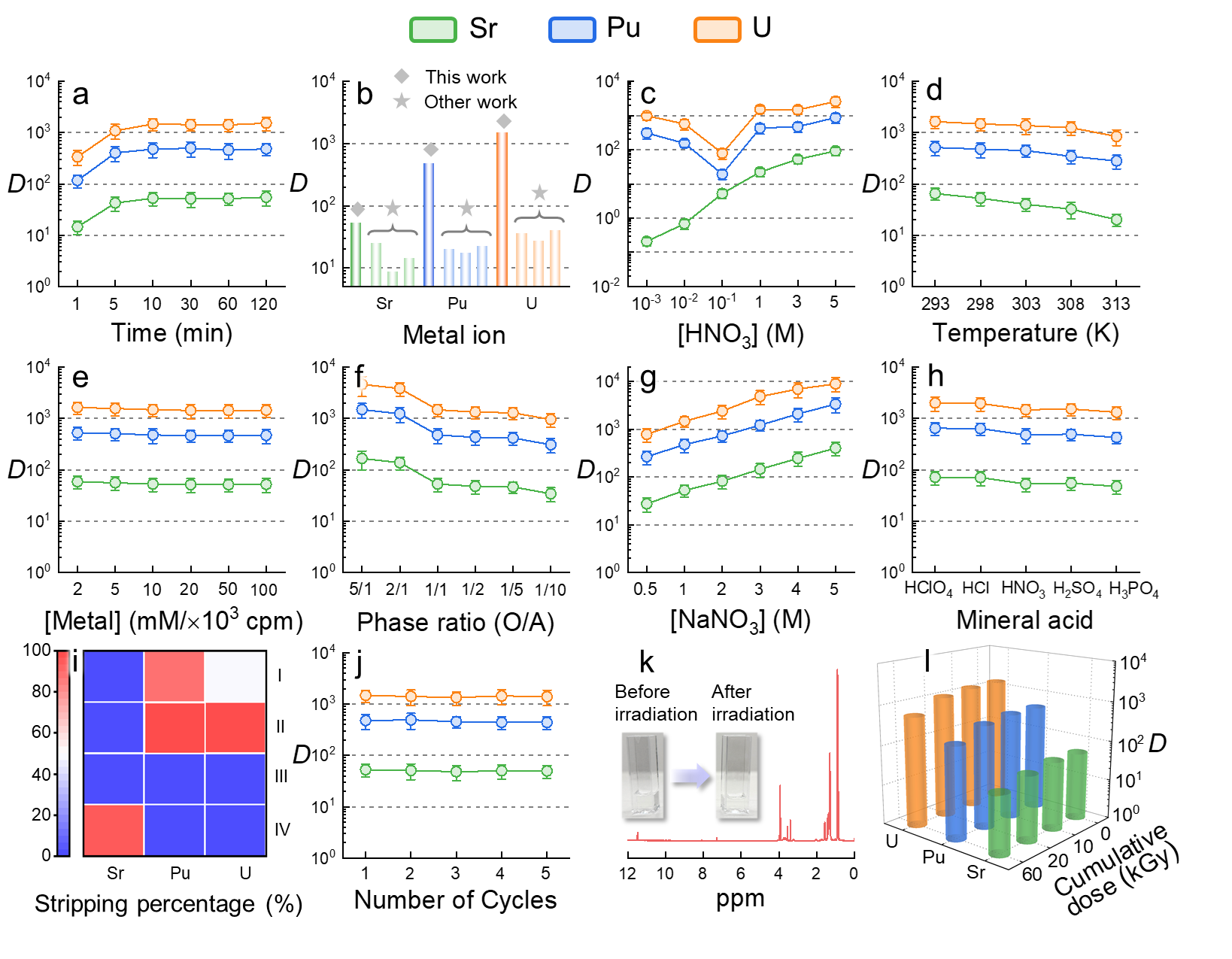


**Figure S11.** Metal ions recovery by DtBuCH18C6-HDEHP EM. Effects of (a) contact time (1-120 min), (c) HNO_3_ concentration (10^-3^-5 M), (d) temperature (293-313 K), (e) metal ion content (2-100 mM) or radioactivity (2-100 × 10^3^ cpm/mL), (f) phase ratio (5/1 to 1/10), (g) NaNO_3_ concentration (0.5-5 M), (h) mineral acid medium, and (l) irradiation dose (0-60 kGy) on the uptake of U, Pu, and Sr. (b) Comparison of recovery performance with representative conventional systems. (i) Stripping and (j) cyclic tests of EM. (k) ^1^H NMR spectrum and image of EM sample induced by cumulative γ-irradiation up to a total dose of 60 kGy.

Standard experimental conditions. Initial organic phase: DtBuCH18C6-HDEHP EM (1/8). Initial aqueous phase: 10 mM U(VI), 10 × 10^3^ cpm/mL Pu(IV), 10 mM Sr(II) mixed with 1 M NaNO_3_ in 3 M HNO_3_. Mixing 10 min at 298.0 ± 0.1 K with a phase ratio (O/A) of 1/1. The applied stripping agents were (I) 0.6 M oxalic acid/H_2_O solution, (II) 1 M Na_2_CO_3_/H_2_O solution, (III) 5 M HNO_3_, and (IV) 10^-3^ M HNO_3_. The γ irradiation dose rate was 5000 Gy/h, with irradiation durations of 2 h, 4 h, and 12 h, resulting in a total cumulative irradiation dose of 10 kGy, 20 kGy, and 60 kGy.


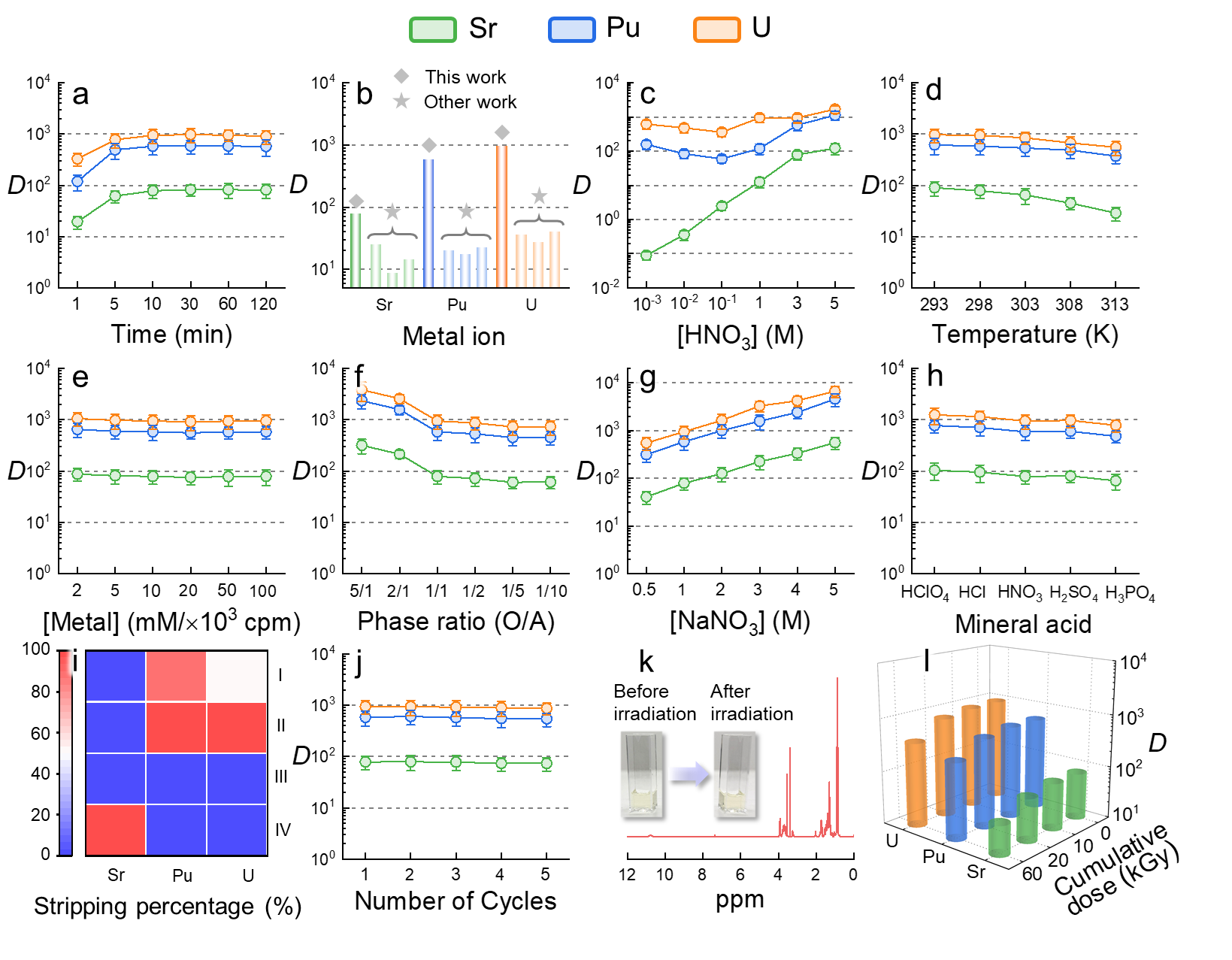


**Figure S12.** Metal ions recovery by DtBuCH18C6-EHEHPA EM. Effects of (a) contact time (1-120 min), (c) HNO_3_ concentration (10^-3^-5 M), (d) temperature (293-313 K), (e) metal ion content (2-100 mM) or radioactivity (2-100 × 10^3^ cpm/mL), (f) phase ratio (5/1 to 1/10), (g) NaNO_3_ concentration (0.5-5 M), (h) mineral acid medium, and (l) irradiation dose (0-60 kGy) on the uptake of U, Pu, and Sr. (b) Comparison of recovery performance with representative conventional systems. (i) Stripping and (j) cyclic tests of EM. (k) ^1^H NMR spectrum and image of EM sample induced by cumulative γ-irradiation up to a total dose of 60 kGy.

Standard experimental conditions. Initial organic phase: DtBuCH18C6-EHEHPA EM (1/2). Initial aqueous phase: 10 mM U(VI), 10 × 10^3^ cpm/mL Pu(IV), 10 mM Sr(II) mixed with 1 M NaNO_3_ in 3 M HNO_3_. Mixing 10 min at 298.0 ± 0.1 K with a phase ratio (O/A) of 1/1. The applied stripping agents were (I) 0.6 M oxalic acid/H_2_O solution, (II) 1 M Na_2_CO_3_/H_2_O solution, (III) 5 M HNO_3_, and (IV) 10^-3^ M HNO_3_. The γ irradiation dose rate was 5000 Gy/h, with irradiation durations of 2 h, 4 h, and 12 h, resulting in a total cumulative irradiation dose of 10 kGy, 20 kGy, and 60 kGy.


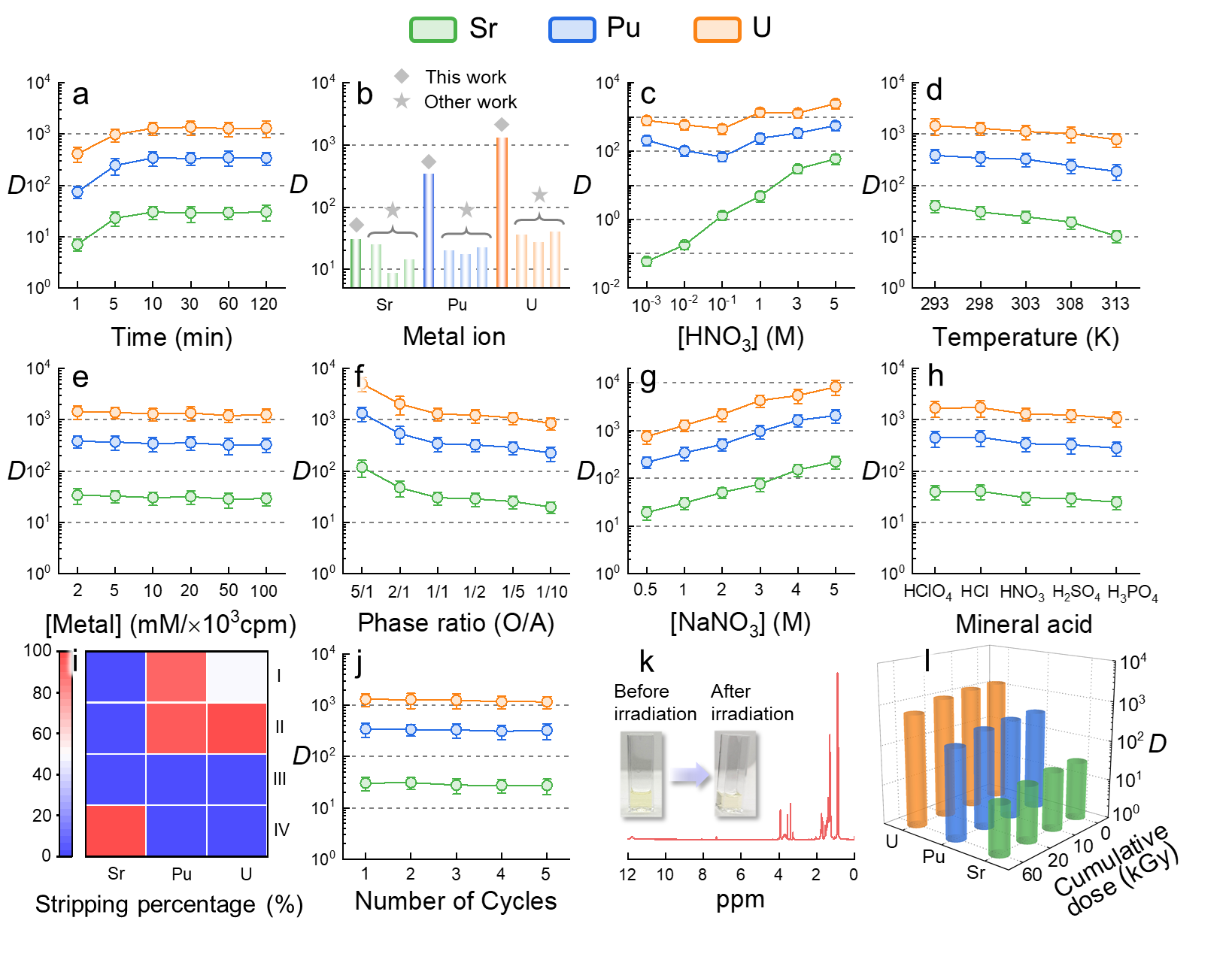


**Figure S13.** Metal ions recovery by DtBuCH18C6-EHEHPA EM. Effects of (a) contact time (1-120 min), (c) HNO_3_ concentration (10^-3^-5 M), (d) temperature (293-313 K), (e) metal ion content (2-100 mM) or radioactivity (2-100 × 10^3^ cpm/mL), (f) phase ratio (5/1 to 1/10), (g) NaNO_3_ concentration (0.5-5 M), (h) mineral acid medium, and (l) irradiation dose (0-60 kGy) on the uptake of U, Pu, and Sr. (b) Comparison of recovery performance with representative conventional systems. (i) Stripping and (j) cyclic tests of EM. (k) ^1^H NMR spectrum and image of EM sample induced by cumulative γ-irradiation up to a total dose of 60 kGy.

Standard experimental conditions. Initial organic phase: DtBuCH18C6-EHEHPA EM (1/8). Initial aqueous phase: 10 mM U(VI), 10 × 10^3^ cpm/mL Pu(IV), 10 mM Sr(II) mixed with 1 M NaNO_3_ in 3 M HNO_3_. Mixing 10 min at 298.0 ± 0.1 K with a phase ratio (O/A) of 1/1. The applied stripping agents were (I) 0.6 M oxalic acid/H_2_O solution, (II) 1 M Na_2_CO_3_/H_2_O solution, (III) 5 M HNO_3_, and (IV) 10^-3^ M HNO_3_. The γ irradiation dose rate was 5000 Gy/h, with irradiation durations of 2 h, 4 h, and 12 h, resulting in a total cumulative irradiation dose of 10 kGy, 20 kGy, and 60 kGy.
